# Effect of Replacing Added Sugars with Sucralose on Gut Microbiome Composition Among Asian Indian Adults in Two 12-week Randomized Controlled Trials

**DOI:** 10.1016/j.cdnut.2025.107600

**Published:** 2025-11-12

**Authors:** Danielle E Haslam, Kuzhandaivelu Abirami, Jacqueline R Starr, Ranjit Unnikrishnan, Jessica Lasky-Su, Rajagopal Gayathri, Kuppan Gokulakrishnan, Valangaiman Sriram Manasa, Eric B Rimm, Ranjit Mohan Anjana, Kamala Krishnaswamy, Frank B Hu, Vasudevan Sudha, Viswanathan Mohan, Shilpa N Bhupathiraju

**Affiliations:** 1 Channing Division of Network Medicine, Department of Medicine, Brigham and Women’s Hospital and Harvard Medical School, Boston, MA, United States; 2 Department of Nutrition, Harvard T.H. Chan School of Public Health, Boston, MA, United States; 3 Department of Foods, Nutrition and Dietetics Research, Madras Diabetes Research Foundation, Chennai, Tamil Nadu, India; 4 Department of Diabetology, Madras Diabetes Research Foundation & Dr. Mohan’s Diabetes Specialities Centre, Chennai, Tamil Nadu, India; 5 Department of Neurochemistry, National Institute of Mental Health and Neuro Sciences, Bengaluru, Karnataka, India; 6 Department of Epidemiology, Harvard T.H. Chan School of Public Health, Boston, MA, United States

**Keywords:** dietary interventions, nonnutritive sweeteners, sucralose, gut microbiome, type 2 diabetes, obesity, Asian Indians

## Abstract

**Background:**

Replacing added sugars with nonnutritive sweeteners, such as sucralose, may help reduce weight gain in adults over time. Because sucralose is primarily excreted in the stool, its consumption could lead to changes in the gut microbiome.

**Objectives:**

We aimed to explore whether replacing sucrose used in beverages with small quantities of sucralose led to gut microbiome changes among Asian Indian adults with type 2 diabetes (T2D) or overweight/obesity (BMI ≥23 kg/m^2^) without T2D.

**Methods:**

In 2 analogous substudies nested within two 12-wk, open-label parallel-arm randomized controlled trials, adults with T2D (n = 49) or overweight/obesity and no T2D (n = 48) were instructed to replace sucrose in their daily coffee and tea with sucralose or to continue their use of sucrose. We examined changes in gut microbiome community structure and taxonomic composition profiled using 16S rRNA sequencing in stool samples collected before and after the 12-wk interventions. The false discovery rate was controlled using the Benjamini-Hochberg method (q < 0.20).

**Results:**

Compared with the control group, the sucralose intervention decreased α diversity (Shannon index: *P* = 0.02; Simpson index: *P* = 0.03) and increased β diversity (*P* = 0.001) in gut microbiome communities of adults with T2D, but not among adults with overweight/obesity (all between-group *P* > 0.05). Among 185 genera tested in the T2D trial, compared with the control, relative abundances of 14 primarily sugar-fermenting or short-chain fatty-acid-producing *Firmicutes* bacteria in the *Lachnospiracae* family were reduced, whereas *Enterococcus* and *Pediococcus* increased during the intervention (q < 0.20). In contrast, adults with overweight/obesity and no T2D showed no similar changes.

**Conclusions:**

Replacing daily sucrose added to coffee and tea with sucralose resulted in changes in gut microbiome community structure and taxonomic composition among Asian Indian adults with T2D, but not those with overweight/obesity and no T2D. Further studies are needed to understand potential health implications and the underlying drivers of these gut microbiome changes.

Clinical Trial Register No. (India Trial Register): CTRI/2021/04/032686, CTRI/2021/04/032809.

## Introduction

Higher consumption of added sugars, particularly from beverages, has consistently been linked with increased weight gain and risk for type 2 diabetes (T2D) [1]. In India, an estimated 11.4% of individuals have diabetes, and 28.6% of individuals have obesity [2]. Over 60% of individuals living in India consume tea daily, and ∼80% of these individuals drink their tea with added sugar [3]. This proportion appears to be consistent among individuals with T2D, based on a recent study in which >50% of individuals in a free-living urban population with T2D reported consuming added sugar in their tea and coffee [4]. Thus, strategies to help reduce the amount of sugar added to these beverages may be fruitful to help curb the rising burden of T2D in India [2,5], which also occurs at a younger age in this ethnic group [6].

Recent studies have demonstrated that replacement of added sugars with nonnutritive sweeteners, such as sucralose, may help reduce weight gain in adults over time [7,8]. Thus, the American Diabetes Association recently recommended that nonnutritive sweeteners can be an acceptable replacement for added sugars, in moderation, if their use helps individuals reduce their overall calorie and carbohydrate intake [9]. Conversely, the WHO recently recommended against the use of nonsugar sweeteners to control body weight or reduce risk of noncommunicable disease [10]. Thus, potential adverse effects of nonnutritive sweeteners are still being explored [7].

Sucralose is a nonnutritive sweetener that is about 600 times sweeter than sucrose [11]. In a recent study among individuals with T2D living in India, replacing added sugar in daily coffee or tea with sucralose for 12 wks resulted in less weight gain with no adverse effects on glycemic or lipid measures [12]. This study agrees with findings from other populations that have also observed reductions in body weight with higher intakes of nonsugar sweeteners [13]. Some previous randomized controlled trials (RCTs) have shown that regular sucralose consumption may cause an impaired insulin response, leading to elevated glycemia [[14], [15], [16]], but evidence is mixed [17,18]. Sucralose is primarily excreted in the stool [19,20], suggesting that it is poorly absorbed and may interact with the gut microbiome [15]. A number of studies have suggested differences in the gut microbiome after sucralose interventions, but studies thus far are short in duration (1–4 wk) with small sample sizes [15,[21], [22], [23], [24]], and no studies have examined differences in gut microbiome after a sucralose intervention among individuals with T2D.

In analogous substudies nested within two 12-wk RCTs among Asian Indian adults with T2D or overweight/obesity and no T2D, we aimed to evaluate the effect of replacing added sugar in tea or coffee with small quantities of sucralose on gut microbial community structure and taxonomic composition.

## Methods

### Study participants

Two isocaloric open-label, parallel-arm RCTs were conducted among (1) 210 individuals with T2D (T2D trial) and (2) 210 individuals without T2D and with overweight or obesity (BMI ≥ 23.0 kg/m^2^) (overweight/obesity trial) at the Madras Diabetes Research Foundation in Chennai, India. In both trials, men and women who were consuming added sugars (sucrose) in their coffee and tea were recruited. Details about the T2D trial have been previously reported [12]. Briefly, participants in the T2D trial were aged 30 to 50 y, had a BMI ≥ 18.5 kg/m^2^, a glycated hemoglobin (HbA1c) of 7–12%, and were on a stable dose of oral hypoglycemic agent(s). In the overweight/obesity trial, participants were aged 25–50 y; had a BMI ≥ 23.0 kg/m^2^, which is the Asian-specific overweight/obesity cut-off [25]; and displayed features of metabolic syndrome or prediabetes. Exclusion criteria included smoking tobacco, acute infections, disorders or diseases that may interfere with the intervention (liver, kidney, thyroid, other endocrine diseases, or possible eating disorder), participants undergoing treatment or medication regimens (e.g. antibiotics), pregnant and lactating women, plans to relocate within 3 y, or long-term travel plans. In the overweight/obesity trial, participants were further excluded if they had a known history of or newly diagnosed T2D.

The primary outcomes in the trials were the change in HbA1c and body weight from baseline to 12 wk in the T2D and overweight/obesity trials, respectively. In 2 gut microbiome substudies within these larger trials, 49 (T2D trial) and 48 (overweight/obesity trial) participants provided stool samples for further analysis (Supplemental Figure 1). No formal sample size calculations were performed for these substudies; rather, the sample size reflects the maximum number of stool samples available to explore the effect of the interventions on changes in the gut microbiome.

### Ethics

The Institutional Ethics Committee at the Madras Diabetes Research Foundation approved both trials before their initiation, and both were conducted in accordance with the Declaration of Helsinki. The trials were registered at the Clinical Trial Registry of India (T2D trial: CTRI/2021/04/032686 and overweight/obesity trial: CTRI/2021/04/032809). Participants provided written informed consent before participation.

### Sucralose intervention

Eligible participants participated in a 1-wk run-in period to evaluate their compliance, and their preference for a pellet, powder, or liquid form of sucralose to be added to their coffee and tea. After a 1-wk washout period, study participants were then randomly assigned (stratified by sex) to the intervention or control arm. Participants in the intervention arm were instructed to replace each teaspoon of added sugar in their coffee or tea with 6 mg of provided sucralose (1 pellet, 1 measured spoonful of powder, or 1 liquid drop) for 12 wks. Participants in the control arm were asked to continue their routine diet. Both groups were instructed to continue their usual physical and lifestyle activities and routine medications. Participants were also instructed not to consume other nonnutritive sweeteners in foods or beverages and not to feast or fast during the study period. Self-reported compliance was collected via 24-h dietary recalls, where the average of 2 recalls collected at screening and baseline was used to estimate baseline diet, and the average of 6 recalls collected during the 12-wk study period was used to estimate 12-wk diet. Nutrient intake estimates were derived using in-house software (EpiNu), where estimates of added sugar (g/d and % energy) and sucralose (mg/d) were used to reflect intervention compliance.

### Sample collection and outcome assessment

At baseline and 12 wks, blood samples were drawn after an overnight fast (10 to 12 h) by venipuncture by a qualified phlebotomist. Plasma glycemic (glucose, insulin, and HbA1c) and serum lipid (total, HDL, and LDL cholesterol and triglyceride concentrations) measures were estimated using standardized protocols that have been previously described [12]. Waist circumference (cm) was measured twice and averaged. BMI was calculated as weight (kg) divided by the square of height (m). Demographics, medical history, and lifestyle factors were obtained via an interviewer-administered questionnaire.

Stool samples were collected at the baseline and 12-wk follow-up visits. All participants were given a stool collection kit (SARSTEDT AG & Co., Feces tube 76×20 mm) and instructions on how to collect and store the sample. The samples were stored in a domestic freezer during transit to the Madras Diabetes Research Foundation, where they were aliquoted and stored at −80°C. Similar methods have demonstrated reliability in previous studies [26].

### Gut microbiome profiling

To assess the bacterial taxa present in the gut microbiome, DNA was extracted from stool samples using QIAamp Fast DNA Stool Mini [Cat No./ID: 51604, QIAGEN] following the manufacturer’s "Fast DNA Stool Mini Handbook" for fast purification of genomic DNA. The V3–V4 region of the 16S rRNA gene was sequenced on an Illumina MiSeq by MedGenome Labs. The sequenced reads were overlapped and stitched to form reads >400 bp using QIIME2 2023.5 [27]. Low-quality sequence reads were excluded from the analysis, and denoising, pair merging, and representative sequence alignment were performed using DADA2 [28]. Taxa were assigned to the SILVA database (version 138) [29], implemented with the QIIME2 feature classifier plugin, and classified at the phyla, order, family, and genus level when possible.

### Changes in gut microbiome community structure

To examine various aspects of microbial community structure, we calculated Bray-Curtis β diversity distances and 3 measures of α diversity: Chao1 (species richness), Shannon index (species richness and evenness), and Simpson index (species evenness and dominance) [30]. To assess the effect of the sucralose intervention on β diversity from baseline to 12 wks separately in each trial, we used the adonis2 and betadisper functions from the *vegan* R package to apply permutational multivariate analysis of variance (PERMANOVA) and permutational analysis of multivariate dispersion (PERMDISP) with 999 permutations on Bray-Curtis dissimilarity matrices. To evaluate differences in the effect of the intervention on pre- and posttreatment changes in β diversity within each trial, we applied a PERMANOVA model including the interaction between intervention group and time (p_group × time_ from a model including group + time + group × time). These differences were illustrated by conducting principal coordinate analyses (PCoA) based on Bray-Curtis distances separately within each intervention group in each trial. To assess the direction of changes in β diversity, we examined the mean distance to the group centroid in multivariate space, where a larger distance reflects increased dispersion and greater community diversity. Next, we examined differences in changes in gut microbiome α diversity between the 2 intervention groups using linear regression models separately in each trial. Paired t-tests were also performed to examine changes in α diversity within groups over time.

### Changes in gut microbiome taxonomic composition

First, we used similar models described for α diversity metrics to examine the effect of the intervention on *Firmicutes: Bacteroides* ratio, a measure that has been positively associated with obesity and metabolic disease [31]. Next, the relative abundances for all phyla- and genus-level taxonomic features were total-sum-scale transformed, and analyses were limited to features detected in >10% of samples. Among the 255 genera detected in the T2D trial, 185 were detected in >10% of samples. Among the 276 genera detected in the overweight/obesity trial (224 of which were also detected in the T2D trial), 194 were detected in >10% of samples. To detect between-group differences in changes in relative abundance of microbial features, we fit linear mixed models including participant ID as a random effect and group, time, group × time interaction, age, and sex as fixed effects using the MaAsLin2 R package [32]. The group × time interaction in this model represents the primary treatment effect, or the between-group difference in relative abundance for each feature. For microbial taxon-specific analyses, we accounted for multiple testing by using the Benjamini-Hochberg procedure, with a false discovery rate (FDR) threshold of 0.20 for q_group × time_. These results were presented in a circular phylogenetic tree that was produced using GraPhlAn (Graphical Phylogenetic Analysis) [33]. Sensitivity analyses were performed restricting to individuals not taking metformin or with obesity (BMI ≥ 25.0 kg/m^2^) in the T2D study.

### Post hoc mediation and secondary analyses

To the extent that the sucralose intervention influences clinically relevant anthropometric measures such as body weight and waist circumference, it is plausible that the effect could be mediated by gut microbiome composition. To test for mediation, we fit linear regression models examining the effect of the sucralose intervention on changes in body weight and waist circumference before and after adjustment for the changes in gut microbiome features that may potentially mediate these associations. We compared their estimated associations and estimated the mediated proportion of the direct effect using the *mediation* R package [34].

In additional post hoc analyses, we examined changes in total energy (kcal), added sugar intake (g and percent energy), carbohydrate (g and percent energy), and sucralose (g) to capture compliance with the intervention in these trials. In the T2D trial, we further examined the association of changes in added sugar and sucralose intake with changes in candidate gut microbial genera utilizing linear mixed-effects regression models including each candidate gut microbial feature as the outcome, participant ID as a random effect, and added sugar or sucralose, time, and added sugar or sucralose intake × time interaction as fixed effects. All statistical analyses were performed using R version 4.2.0 and Python version 3.8.2. There were no missing covariate values. A *P* < 0.05 was used as a cut-off for statistical significance unless otherwise specified.

## Results

### Participant baseline characteristics

Participants in the T2D trial were slightly older and had a higher waist circumference than those in the overweight/obesity trial, whereas mean BMI was similar in both trials at around 29 kg/m^2^ (Table 1). Two-thirds or more participants were female in both trials. Diabetes-related traits were less favorable in the T2D trial compared with the overweight/obesity trial. At baseline, compared with participants in the overweight/obesity trial, participants in the T2D trial had higher mean glucose, insulin, and HbA1c. Baseline characteristics and dietary intakes were similar across the intervention and control groups in both trials, except for total sugar intake, which was higher in the control arm in the overweight/obesity trial compared with the intervention arm (*P* = 0.01).

**TABLE 1:** Baseline characteristics of study participants stratified by sucralose intervention or control arm among individuals with gut microbiome data in the type 2 diabetes and overweight/obesity trials^1^.

	Type 2 diabetes trial^2^				Overweight/obesity trial^2^			
	All (n = 49)	Intervention (n = 25)	Control (n = 24)	P value	All (n = 48)	Intervention (n = 23)	Control (n = 25)	P value
Age (y)	45.2 (5.3)	46.3 (3.3)	44.0 (6.7)	0.13	37.0 (8.6)	35.7 (8.9)	38.2 (8.2)	0.31
Waist circumference (cm)	97.4 (8.0)	96.7 (8.5)	98.1 (7.6)	0.56	94.2 (10.5)	95.4 (12.2)	93.1 (8.8)	0.45
BMI (kg/m^2^)	29.4 (4.5)	29.5 (4.0)	29.3 (5.1)	0.86	29.3 (4.4)	30.2 (4.8)	28.4 (3.8)	0.15
Female (%)	82	84	79	0.95	67	60	72	0.61
Alcohol (% current)	0	0	0	—	2	0	4	0.98
Income (K INR/mo)	19 (15)	16 (10)	22 (18)	0.19	21 (19)	20 (16)	21 (21)	0.86
Fasting glucose (mg/dL)	162 (53)	156 (45)	168 (61)	0.46	83.3 (10.9)	81.8 (10.0)	84.7 (11.8)	0.37
Fasting insulin (μIU/mL)	20.3 (7.9)	18.9 (8.8)	21.3 (9.0)	0.78	16.4 (8.1)	17.3 (9.1)	15.6 (7.1)	0.46
Glycated hemoglobin (%)	9.0 (1.1)	8.9 (1.0)	9.1 (1.2)	0.42	5.7 (0.4)	5.7 (0.4)	5.6 (0.4)	0.93
Diabetes duration (y)	3.8 (2.2)	3.9 (2.0)	3.8 (2.4)	0.86	—	—	—	—
Diabetes medication (% yes)	92	84	100	0.13	0	0	0	—
Metformin use (% yes)	53	48	58	0.66	0	0	0	—
Daily Dietary Intakes								
Energy (kcal)	1424 (335)	1443 (380)	1405 (288)	0.70	1609 (359)	1565 (355)	1649 (365)	0.42
Carbohydrate (% energy)	59.9 (6.1)	60.4 (6.7)	59.4 (5.5)	0.55	58.9 (6.4)	60.1 (6.6)	57.8 (6.2)	0.22
Total sugar (% energy)	8.0 (3.6)	7.3 (3.5)	8.8 (3.6)	0.15	8.2 (2.9)	7.1 (2.5)	9.2 (2.9)	0.01
Added sugar (g)	15.7 (10.7)	13.7 (10.2)	17.9 (11.1)	0.17	17.2 (11.1)	18.1 (9.7)	16.4 (12.5)	0.63
Fiber (g)	23.9 (7.8)	23.5 (8.2)	24.3 (7.4)	0.72	22.3 (6.0)	23.2 (6.7)	21.5 (5.2)	0.34
Protein (% energy)	11.3 (1.3)	11.6 (1.5)	11.0 (1.0)	0.11	12.7 (1.5)	12.8 (1.4)	12.6 (1.6)	0.60
Saturated fat (% energy)	6.6 (2.4)	6.5 (2.3)	6.7 (2.6)	0.68	7.4 (2.5)	6.9 (2.1)	7.8 (2.9)	0.26
Monunsaturated fat (% energy)	5.4 (1.7)	5.2 (1.6)	5.6 (1.9)	0.45	6.7 (1.5)	6.6 (1.8)	6.8 (1.3)	0.77
Polyunsaturated fat (% energy)	9.8 (2.5)	9.6 (2.6)	9.9 (2.3)	0.71	11.5 (1.0)	11.7 (2.4)	11.4 (1.6)	0.61

Abbreviations: HbA1c, glycated hemoglobin; INR, Indian Rupee; kcal, kilocalories.

1 Values are means (SD) for continuous variables and % for categorical variables. P values are derived from a 2-sample t-test comparing the mean difference between the intervention and control arms for continuous variables and chi-squared tests for categorical variables.

2 Individuals included in the type 2 diabetes trial had a type 2 diabetes diagnosis at baseline, and individuals included in the overweight/obesity trial had a BMI ≥ 23.0 kg/m^2^ and did not have a type 2 diabetes diagnosis at baseline.

### Effect of the intervention on changes in gut microbiome community structure

**FIGURE 1:**
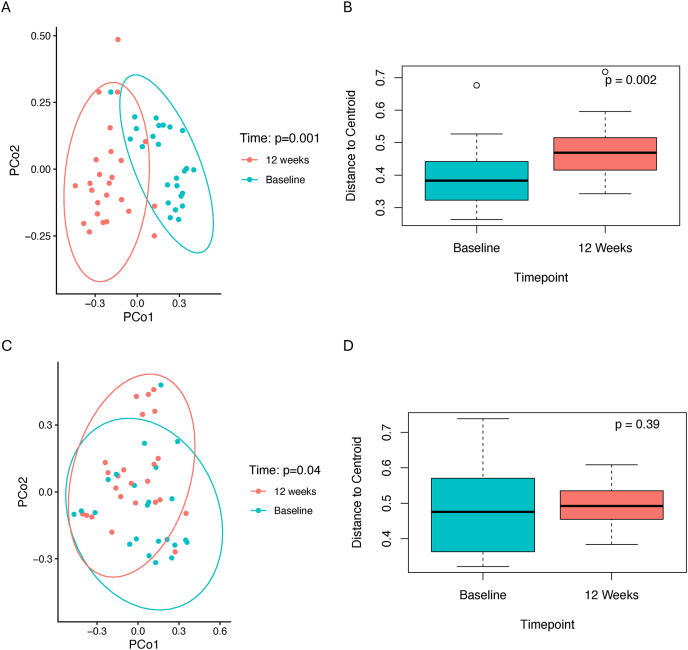
In the type 2 diabetes trial, differences in gut microbiome β diversity are displayed through principal coordinate analyses (PCoA) based on Bray-Curtis distances by time point in the (A) sucralose arm and (C) control arm. P values are derived from PERMANOVA models examining changes in β diversity within groups from baseline to 12 weeks. Boxplots additionally show the mean distance of each sample’s Bray-Curtis distance from the group centroid by time point in the (B) sucralose arm and (D) control arm. P values are derived from t-tests comparing means distances at each time point within each arm. Boxes indicate the median and interquartile range, and whiskers indicate 1.5 times the interquartile range.

**FIGURE 2:**
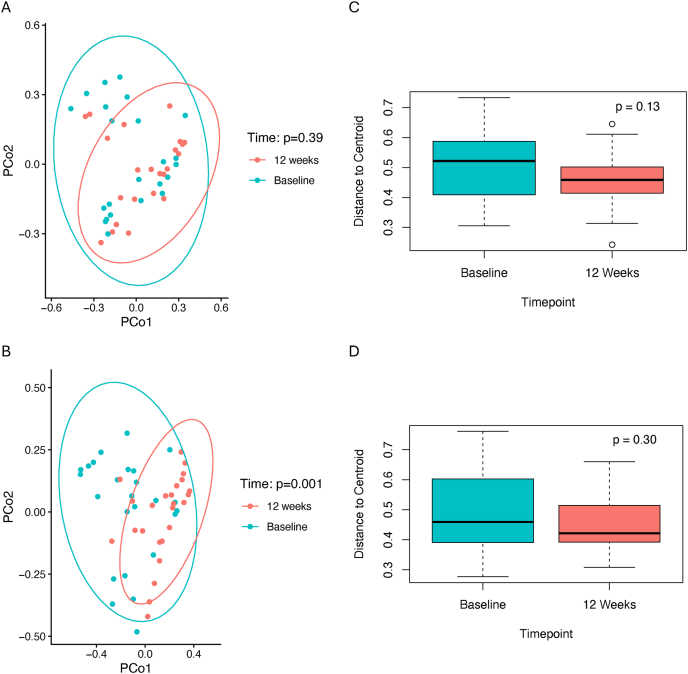
In the overweight/obesity trial, differences in gut microbiome β diversity are displayed through principal coordinate analyses (PCoA) based on Bray-Curtis distances by time point in the (A) sucralose arm and (B) control arm. P values are derived from PERMANOVA models examining changes in β diversity within groups from baseline to 12 weeks. Boxplots additionally show the mean distance of each sample’s Bray-Curtis distance from the group centroid by time point in the (C) sucralose arm and (D) control arm. *P* values are derived from t-tests comparing mean distances at each time point within each arm. Boxes indicate the median and interquartile range, and whiskers indicate 1.5 times the interquartile range.

Pre- and posttreatment gut microbiome β diversity measures differed by treatment gro in the T2D trial (p_group x time_=0.007), but not the overweight/obesity trial (*P*_group x time_ = 0.50). In the T2D trial, pre- and posttreatment increases in β diversity were greater in the sucralose arm (PERMANOVA: *P* = 0.001 [Figure 1A]; PERMDISP: *P* = 0.002 [Figure 1B]) than in the control arm (PERMANOVA: *P* = 0.04 [Figure 1C]; PERMDISP: *P* = 0.39 [Figure 1D]). Conversely, in the overweight/obesity trial, pre- and posttreatment differences in β diversity were greater in the control arm (*P* =0.001; Figure 2B) than in the sucralose arm (*P* = 0.39; Figure 2A), yet beta dispersion estimates (PERMDISP) were similar pre- and posttreatment in both the intervention (*P* = 0.13; Figure 2C) and control (*P* = 0.30; Figure 2D) groups.

Across the 2 substudies and treatment groups and by any metric (Chao1, Shannon index, or Simpson index), α diversity was either similar or higher at 12 wks compared with baseline in all the groups except in the T2D trial intervention group, in which α diversity declined over the 12 wks (between-group difference [95% CI]: Chao1, −530 [−1338, 278], *P* = 0.19; Shannon Index, −0.83 [−1.49, −0.17], *P* =0.02; Simpson index, −0.08 [−0.16, −0.01], *P* = 0.03) (Table 2). Sensitivity analyses, additionally adjusting for metformin use, yielded similar results (data not shown).

**TABLE 2:** Change in overall gut microbiome community measures among study participants with type 2 diabetes (n =49) or overweight/obesity (n=48) randomized into the sucralose intervention or control arm^1^.

Variables	Control arm				Intervention arm				Between-group difference (95% CI)	Between-group P value
	Baseline	End of 12 wk	Change	Within-group P value	Baseline	End of 12 wk	Change	Within-group P value		
Type 2 diabetes trial										
Chao1	2599 (1465)	2725 (1213)	126 (1349)	0.65	2830 (1454)	2426 (816)	−404 (1457)	0.18	−530 (−1338, 278)	0.19
Shannon index	7.13 (1.21)	7.09 (1.13)	−0.04 (1.25)	0.89	7.67 (0.96)	6.80 (0.94)	−0.87 (1.05)	0.0004	−0.83 (−1.49, −0.17)	0.02
Simpson index	0.82 (0.15)	0.84 (0.08)	0.02 (0.15)	0.55	0.87 (0.06)	0.80 (0.09)	−0.07 (0.10)	0.005	−0.08 (−0.16, −0.01)	0.03
Firmicutes:Bacteroides	1.72 (2.34)	2.70 (1.90)	0.98 (3.22)	0.15	0.81 (0.70)	2.92 (2.21)	2.19 (2.08)	<0.0001	1.21 (−0.37, 2.79)	0.13
Overweight/obesity trial										
Chao1	2822 (1395)	3310 (856)	488 (1778)	0.18	2596 (1140)	3177 (958)	581 (1350)	0.05	93 (−830, 1016)	0.84
Shannon index	7.36 (1.20)	7.44 (0.77)	0.08 (1.22)	0.73	7.06 (1.03)	7.48 (0.73)	0.42 (1.26)	0.12	0.34 (−0.38, 1.06)	0.35
Simpson index	0.80 (0.13)	0.87 (0.05)	0.07 (0.15)	0.02	0.80 (0.11)	0.86 (0.05)	0.06 (0.13)	0.05	−0.02 (−0.10, 0.06)	0.65
Firmicutes:Bacteroides	1.58 (2.11)	3.43 (3.00)	1.86 (3.38)	0.01	2.29 (2.24)	2.68 (2.04)	0.47 (3.32)	0.52	−1.39 (−3.36, 0.58)	0.16

1 Data presented as mean ± SD, unless otherwise noted. Chao1, Shannon index, and Simpson index are all measures of α diversity. Individuals included in the type 2 diabetes trial had a type 2 diabetes diagnosis at baseline, and individuals included in the overweight/obesity trial had a BMI ≥ 23.0 kg/m^2^ and did not have a type 2 diabetes diagnosis at baseline.

### Effect of the intervention on changes in gut microbial taxonomic composition

No treatment-group differences in changes in relative abundance of bacterial phyla met the FDR threshold in either trial (Supplemental Figure 2). In the T2D trial, the *Firmicutes:Bacteroides* ratio increased from baseline to 12 wks more steeply in the intervention group (mean [SD] change: 2.19 [2.08], *P* < 0.0001) than in the control group (mean [SD] change: 0.98 [3.22] *P* = 0.15; between-group difference, *P* = 0.13) (Table 2). Conversely, in the overweight/obesity trial, the *Firmicutes:Bacteroides* ratio increased from baseline to 12 wk more steeply in the control group (mean [SD] change: 1.86 [3.38], *P* = 0.01) than in the intervention group (mean [SD] change: 0.47 [3.32], *P* = 0.52; between-group difference, *P* = 0.16).

**FIGURE 3:**
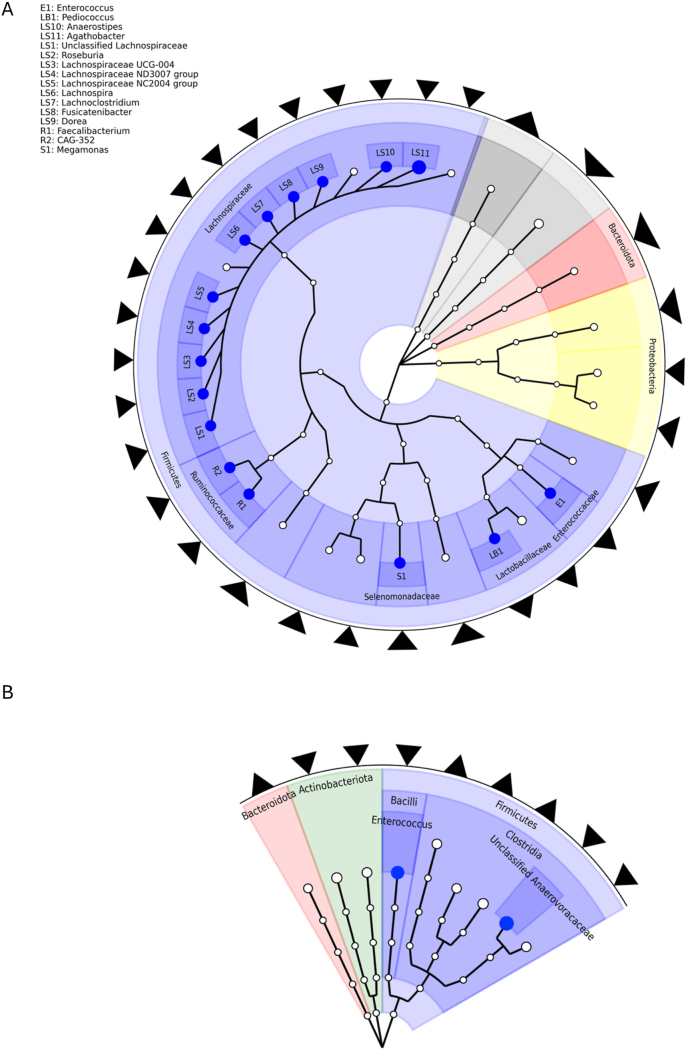
Effects of a 12-week sucralose intervention on gut microbial genera among individuals in the (A) type 2 diabetes trial or (B) overweight/obesity trial are displayed within a circular phylogenetic generated using GraPhlan2 [33]. Only genera with *P* < 0.05 are displayed in this figure to enhance visualization. A blue colored node indicates a genus with q < 0.20. Arrows around the edge of the circular phylogenetic tree indicate the direction of the β coefficient. *P* values and β coefficients are derived from the group × time interaction in a linear mixed model including each gut microbial genus relative abundance as the outcome, participant ID as a random effect, and group, time, group × time interaction, age, and sex as fixed effects.

Compared with the control group, decreases in the relative abundance of 14 *Firmicutes* bacteria (β_group x time_ ranging from −0.82 to −2.30) were larger in the sucralose intervention group in the T2D trial (all q_group x time_ < 0.20) (Figure 3A; Supplemental Table 1). Most of these bacteria belonged to the *Lachnospiraceae* or *Ruminococcaceae* families, and the genera they belonged to tend to include short-chain fatty acid-producing (e.g. *Agothobacter*, *Lachnoclostridium*, *Lachnospira*, *Megamonas*, *Faecalibacterium*, *Roseburia*) and sugar-fermenting (e.g. *Fusicatenibacter*, *Dorea*, *Faecalibacterium*) bacteria. Conversely, bacteria in the *Enterococcus (*β_group x time_ [SD]: 2.67 [0.81]) and *Pediococcus* (2.42 [0.82]) genera, both of which are in the Lactobacillales order and often include carbohydrate-fermenting bacteria, displayed larger increases in the intervention group compared with the control group in the T2D trial (q_group x time_ = 0.17 for both). In the overweight/obesity trial, only 2 *Firmicutes* genera (Unclassified *Anaerovoracaceae*: β_group x time_ [SD]: −0.53 [0.17], q_group x time_ = 0.13; *Enterococcus*: β_group x time_ [SD]: −1.85 [0.64], q = 0.18) displayed larger decreases in abundance in the intervention compared with the control group and met the FDR threshold (Figure 3B; Supplemental Table 2). Among approximately half of the participants in the T2D trial not taking metformin, there were some differences in the effect of the intervention on gut microbial taxa, but the genera displaying the largest differences in abundance were largely similar (Supplemental Table 3). Results were also similar among participants with obesity (BMI ≥ 25.0 kg/m^2^) in the T2D trial (Supplemental Table 4).

### Post hoc mediation and secondary analyses

In the T2D trial, participants in the sucralose intervention arm displayed larger mean (SD) decreases in body weight (−1.1 [1.3] kg, *P* = 0.0004) and waist circumference (−0.9 [1.7] cm, *P* = 0.01) compared with the control group (Δ body weight: −0.2 [1.7] kg, *P* = 0.60; Δ WC: −0.3 [2.2] cm, *P* = 0.52), with a between-group *P* = 0.05 for body weight and *P* = 0.26 for waist circumference (Supplemental Table 5). These results were negligibly changed after further adjustment for changes in gut microbial features (Supplemental Table 6). Adjusting for change in the Simpson index slightly attenuated the association between the intervention and changes in body weight, but the confidence intervals for the proportion of the direct effect mediated through the gut microbiome were wide (proportion mediated [95% CI], 32 [−16, 192], *P* = 0.15), suggesting that we had very limited power to evaluate potential mediation. No significant differences in anthropometric measures were observed between or within groups in the overweight/obesity trial (Supplemental Table 7).

Participants reported decreases in added sugar and increases in sucralose intake during the intervention in both the T2D (Δ added sugar: −12.5 [9.7] g, *P* < 0.0001; Δ sucralose: 13.1 [4.0] mg, *P* < 0.0001; Supplemental Table 5) and overweight/obesity (Δ added sugar: −17.6 [9.4] g, *P* < 0.0001; Δ sucralose: 13.0 [5.7] mg, *P* < 0.0001; Supplemental Table 7) trials. Changes in added sugar (7/16 genera; *P* < 0.05) and sucralose (8/16 genera; Shannon Index; *P* < 0.05) intake during the intervention in the T2D trial were nominally associated with changes in gut microbial features during the intervention (Supplemental Tables 8 and 9).

## Discussion

In this study, we examined changes in the gut microbiome after an intervention replacing sugar in coffee and tea with sucralose among individuals with T2D or overweight/obesity. Intervention-related changes in the gut microbiome occurred among individuals with T2D (most of whom were overweight or obese), but not in individuals without T2D and with overweight or obesity. In the T2D trial, we observed changes in both α- and β-diversity and an increase in *Firmicutes: Bacteroides* ratio in the intervention group, but not the control group. The relative abundance of 14 *Firmicutes* bacterial genera decreased and 2 *Firmicutes* bacterial genera increased after the sucralose intervention in the T2D trial. Many of the bacteria reduced during the intervention in the T2D trial belonged to the *Lachnospiracae* family, a family enriched among individuals with T2D [[35], [36], [37]] that are often sugar-fermenting or short-chain fatty acid-producing. In contrast, in the overweight/obesity trial, we detected no significant differences in overall gut microbiome community structure by intervention group and minimal differences in changes among individual taxa.

This is the first report on the impact of a 12-wk sucralose intervention on the gut microbiome among middle-aged adults with T2D or overweight/obesity. Previous interventions were conducted among healthy young adults, and most were short in duration (1 to 2 wk). In one study, significant differences in gut microbial community composition were observed after a 2-wk sucralose intervention among healthy young adults [15], yet no differences were observed in 2 other short-term sucralose interventions [22,23]. In another 10-wk sucralose intervention among healthy young adults, the authors observed some differences in gut microbial species following the intervention, including a significant decrease in *Lactobacillus acidophilus*, which is a sugar-consuming Firmicutes species [24]. No other changes in gut microbial abundance were consistent between our study and previous studies.

Underlying metabolic and gut microbiome differences in individuals with and without T2D may explain the differential effect of sucralose consumption on the gut microbiome in the 2 trials. Personalized responses to sucralose consumption have been reported in several studies [38]. The reported large interindividual variability in excreted sucralose levels [15,20,39,40] suggests that differences in the gut microbiome may drive personalized responses to sucralose intake. Individuals with T2D also have distinct gut microbiome profiles [[35], [36], [37]] and often take metformin, which is known to alter gut microbiome profiles [41]. Previous studies suggest that *Lachnospiracae* family bacteria are enriched among individuals with T2D [[35], [36], [37]]. Thus, individuals in the T2D trial likely started with higher abundances of *Lachnospiracae* bacteria than individuals in the overweight/obesity trial, leaving them with greater capacity for decline during the intervention. This could explain why 11 genera from the *Lachnospiracae* family decreased in abundance in the T2D trial but not the overweight/obesity trial. The small sample sizes in these 2 substudies make it difficult to explore which factors could be driving the differences in the effect of the intervention on the gut microbiome composition in these 2 trials, but future research could explore differential effects of sucralose based on baseline gut microbiome and medication use.

Besides underlying metabolic differences in people with and without T2D, differences in background diet and intervention compliance could also explain differences in the effect of the intervention in the 2 trials. For example, the differential effects of sucralose consumption on glucose responses reportedly depend on concurrent carbohydrate consumption [42], whose consumption patterns may differ among those with and without T2D. Due to the nature of this substitution trial, it is difficult to distinguish whether the effect of the intervention is due to the addition of dietary sucralose, the reduction in dietary added sugar, or other unintended dietary changes that may have occurred during the intervention. In the T2D trial, there were no significant differences in self-reported baseline or changes in dietary intakes by intervention arm, besides the expected differences in sucralose and added sugar intake. However, in the overweight/obesity trial, the control group reported higher carbohydrate intake at baseline and larger increases in dietary fiber during the intervention compared to the intervention group. Thus, it is possible that the small increase in dietary fiber intake in the control arm could have offset or led to gut microbial changes in the sucralose intervention arm in the overweight/obesity trial. The observed decreases in the relative abundance of sugar-fermenting bacteria after the intervention in the T2D trial suggest that gut microbial changes during the intervention may be a result of a decrease in the availability of sugar as a substrate for these bacteria, rather than a result of the addition of sucralose to the diet. However, our secondary analyses found that continuous sucralose, but not added sugar, intake was associated with lower α diversity in the T2D trial, indicating that higher sucralose could also be a driver of the observed changes in the gut microbiome. This could be related to potential bacteriostatic effects of sucralose that have been reported in in vitro studies [43,44]. Animal studies show mixed evidence for whether sucralose consumption in mice leads to an inhibition of gut microbial species growth [[45], [46], [47]]. There may be particular taxa that are more susceptible to sucralose’s bacteriostatic effect, an area for more research [43], but our secondary analyses were inconclusive.

Although this study is the largest and longest investigating the effects of a sucralose intervention on changes in gut microbiome composition among individuals with T2D or overweight/obesity, the sample size is still small. The small sample size increases the likelihood of false positives and false negatives, yet the biological plausibility of our findings increases the likelihood that some of our findings may be true positives. The 16S rRNA sequencing technology provides comprehensive taxonomic profiling of the gut microbiome up to the genus level, but limits our ability to reliably capture species-level classification and understand the functional potential of the gut microbiome. We also did not have an objective measure of intervention compliance and relied on self-reported intakes to estimate compliance during the trials. One challenge of this study is that participants were asked to replace sugar in only coffee or tea with sucralose. Therefore, it is possible that participants who consume several cups of coffee or tea per day would receive a greater dose of sucralose compared with participants who consume fewer cups of coffee or tea. It is not clear if effects would be different if participants replaced all added sugar in their diet, in addition to that added to coffee and tea, with sucralose. In general, the dose of sucralose was relatively low compared with other studies, where participants consumed an average of 13 mg/d. Future studies with larger sample sizes and larger doses, aiming to understand personalized responses to sucralose interventions, will be fruitful in understanding what may be driving the observed differential effects of sucralose on gut microbiome composition among individuals with overweight/obesity or T2D in this study. In addition, although we only tested the effects of sucralose, the effect of other nonnutritive sweeteners on metabolic and gut health remains understudied, especially among Asian Indians who have a disproportionately high burden of cardiometabolic diseases [48].

The potential health effects of the observed changes in gut microbial bacteria, particularly the decreases in sugar-consuming bacteria and increases in β-diversity, in the T2D trial are not fully understood. However, decreases in α-diversity, lower *Firmicutes: Bacteroides* ratio, and lower abundance of short-chain fatty-acid-producing bacteria have all been associated with poor gut health [[49], [50], [51]]. Although reductions in these measures could point to potential harmful effects of dietary sucralose on the gut microbiome among individuals with T2D, they were not correlated with metabolic worsening but rather with benefit. Moreover, we did not observe similar effects of the sucralose intervention on these gut microbial measures in a separate trial among individuals with overweight/obesity and no T2D. Thus, the clinical significance of these findings remains unclear. Further investigations to disentangle the effect of adding sucralose or reducing added sugar in the diet on the gut microbiome are needed. These studies could include further investigation of gut microbial responses to sucralose consumption based on underlying participant characteristics (e.g., presence of T2D, medication use, overweight, or obesity) or further characterization of changes in gut microbiome functional potential to reveal personalized responses to sucralose interventions. Thus, larger, long-term trials leveraging the latest advances in technology to characterize gut microbiome response to sucralose consumption are warranted.

## Author contributions

The authors’ responsibilities were as follows – DEH, VS, VM, and SNB designed the research; KA, RMA, VS, and VM conducted the research; DEH analyzed the data; and DEH, JRS, VM, and SNB wrote the paper. DEH had primary responsibility for the final content. All authors provided input on data interpretation, critically revised the manuscript, and read and approved the final manuscript.

## Data availability

Data described in the manuscript, code book, and analytic code will be made available upon request, pending reasonable request.

## Funding

The study was funded by M/S Zydus Wellness, which also provided the tabletop sweetener in 3 different formats (pellet, liquid, and powder) containing sucralose for use in the study. The sponsors had no role in the study conduct or data analysis. This work was also supported by the National Institutes of Health: K01DK136968 (D.E.H.).

## Conflict of interest

Shilpa N. Bhupathiraju is an Editor for Current Developments in Nutrition and played no role in the Journal’s evaluation of the manuscript. The authors report no conflicts of interest.
