# Supplementary material for: Effect of Replacing Added Sugars with Sucralose on Gut Microbiome Composition Among Asian Indian Adults in Two 12-week Randomized Controlled Trials
Source: Curr Dev Nutr. 2025 Nov 12;9(12):107600. doi: 10.1016/j.cdnut.2025.107600 (PMC12731820; doi:10.1016/j.cdnut.2025.107600)
Supplement: Multimedia component 2 [file mmc2.docx]

**Supplementary Materials**

Haslam et al. Effect of replacing added sugars with sucralose on gut microbiome composition among Asian Indian adults in two 12-week randomized controlled trials

**Supplemental Figure 1**. Participant flow chart for A) type 2 diabetes trial and B) overweight/obesity trial

A)

**Type 2 Diabetes Trial:** Screening for participants (n=300)

Control dropout: n= 17

End of study participants in control: n = 88

Stool samples provided & gut microbiome analysis

**Control statistical analysis:**

**n= 24**

Stool samples provided & gut microbiome analysis

**Intervention statistical analysis:**

**n= 25**

Intervention dropout: n= 14

End of study participants in intervention: n= 91

**Control group (n= 105)**

- Continued to consume added sugar (sucrose) in coffee/ tea daily for 12 weeks.
- Advised to not change other lifestyle factors including diet and physical activity.

**Intervention group (n= 105)**

- Replaced added sugar (~3 tsp.) in coffee/tea with sucralose daily for 12 weeks.
- Advised to not change other lifestyle factors including diet and physical activity.

Enrollment, washout & randomization

(n= 210)

B)

**Overweight/Obesity Trial:** Screening for participants (n=328)

Stool samples provided & gut microbiome analysis

**Control statistical analysis:**

**n= 25**

Control dropout: n= 4

End of study participants in control n = 101

Stool samples provided & gut microbiome analysis

**Intervention statistical analysis:**

**n= 23**

Intervention dropout: n= 8

End of study participants in intervention: n= 97

**Control group (n= 105)**

- Continued to consume added sugar (sucrose) in coffee/ tea daily for 12 weeks.
- Advised to not change other lifestyle factors including diet and physical activity.

**Intervention group (n= 105)**

- Replaced added sugar (~3 tsp.) in coffee/tea with sucralose daily for 12 weeks.
- Advised to not change other lifestyle factors including diet and physical activity.

Enrollment, washout & randomization

(n= 210)

**Supplemental Figure 2**. Mean gut microbial phyla relative abundances by sucralose intervention arm and time in the A) type 2 diabetes trial and B) overweight/obesity trial. All comparisons p > 0.05.

A)

B)

**Supplemental Table 1**. Effect of a 12-week sucralose intervention on gut microbial genera among individuals in the type 2 diabetes trial (See supplemental excel file: “SupplementalTables_FINAL_Haslam_2025.xlsx”)

**Supplemental Table 2**. Effect of a 12-week sucralose intervention on gut microbial genera among individuals in the overweight/obesity trial (See supplemental excel file: “SupplementalTables_FINAL_Haslam_2025.xlsx”))

**Supplemental Table 3**. Effect of a 12-week sucralose intervention on gut microbial genera among individuals not taking metformin in the type 2 diabetes trial (See supplemental excel file: “SupplementalTables_FINAL_Haslam_2025.xlsx”)

**Supplemental Table 4**. Effect of a 12-week sucralose intervention on gut microbial genera among individuals with obesity (BMI ≥ 25.0 kg/m^2^) in the type 2 diabetes trial (See supplemental excel file: “SupplementalTables_FINAL_Haslam_2025.xlsx”)

**Supplemental Table 5.** Change in anthropometric and diet measures among study participants randomized into the sucralose intervention or control arm in a subset of participants with gut microbiome data in the type 2 diabetes trial (n=49)^*^

| **Variables** | **Control group (n=24)** | | | | **Intervention group (n=25)** | | | | **Between-group difference in change**  **(95% CI)** | **Between-group**  **p-value** |
| --- | --- | --- | --- | --- | --- | --- | --- | --- | --- | --- |
|  | **Baseline** | **End of 12 weeks** | **Change** | **Within-group**  **p-value** | **Baseline** | **End of 12 weeks** | **Change** | **Within-group**  **p-value** |  |  |
| Body weight, kg | 70.0 ± 12.3 | 69.8 ± 12.9 | -0.2 ± 1.7 | 0.60 | 69.5 ± 13.0 | 68.4 ± 12.7 | -1.1 ± 1.3 | 0.0004 | -0.9 (-1.7, -0.01) | 0.05 |
| Body mass index, kg/m^2^ | 29.3 ± 5.1 | 29.2 ± 5.2 | -0.1 ± 0.7 | 0.53 | 29.5 ± 4.0 | 29.0 ± 3.9 | -0.4 ± 0.6 | 0.0006 | -0.4 (-0.7, 0.02) | 0.06 |
| Waist circumference, cm | 98.1 ± 7.6 | 97.8 ± 8.0 | -0.3 ± 2.2 | 0.52 | 96.7 ± 8.5 | 95.8 ± 8.4 | -0.9 ± 1.7 | 0.01 | -0.6 (-1.8, 0.5) | 0.26 |
| Total Energy, Kcal | 1405 ± 288 | 1481 ± 282 | 76 ± 267 | 0.18 | 1443 ± 380 | 1425 ± 281 | -18 ± 281 | 0.75 | -94 (-252, 64) | 0.24 |
| Carbohydrate, g | 224 ± 45.8 | 227 ± 41.8 | 2.7 ± 40.6 | 0.75 | 229 ± 52.2 | 210 ± 32.3 | -19.3 ± 51.4 | 0.07 | -21.9 (-48.6, 4.8) | 0.11 |
| Carbohydrate, % energy | 59.4 ± 5.5 | 57.7 ± 5.0 | -1.7 ± 3.1 | 0.01 | 60.4 ± 6.7 | 57.7 ± 4.2 | -2.8 ± 6.4 | 0.04 | -1.1 (-4.0, 1.8) | 0.46 |
| Total Sugar, g | 30.1 ± 11.9 | 32.5 ± 8.3 | 2.4 ± 11.2 | 0.31 | 25.6 ± 10.8 | 13.5 ± 4.0 | -12.1 ± 11.4 | <0.0001 | -14.5 (-21.0, -8.0) | <0.0001 |
| Total Sugar, % energy | 8.8 ± 3.6 | 8.8 ± 2.3 | 0.08 ± 3.1 | 0.90 | 7.3 ± 3.5 | 3.9 ± 1.3 | -3.4 ± 3.6 | <0.0001 | -3.5 (-5.5, -1.6) | 0.0007 |
| Added Sugar, g | 17.9 ± 11.1 | 18.7 ± 9.3 | 0.9 ± 8.7 | 0.64 | 13.7 ± 10.2 | 1.2 ± 3.3 | -12.5 ± 9.7 | <0.0001 | -13.3 (-18.6, -8.0) | <0.0001 |
| Added Sugar, % energy | 4.7 ± 3.0 | 4.7 ± 3.5 | -0.04 ± 2.1 | 0.92 | 3.6 ± 2.7 | 0.3 ± 0.8 | -3.3 ± 2.5 | <0.0001 | -3.3 (-4.6, -2.0) | <0.0001 |
| Fiber, g | 24.3 ± 7.4 | 22.9 ± 5.3 | -1.4 ± 7.2 | 0.34 | 23.5 ± 8.2 | 21.7 ± 4.6 | -1.8 ± 6.6 | 0.18 | -0.4 (-4.4, 3.6) | 0.84 |
| Protein, g | 42.5 ± 10.0 | 46.0 ± 10.8 | 3.5 ± 10.2 | 0.11 | 44.8 ± 13.3 | 44.9 ± 10.6 | 0.1 ± 9.7 | 0.95 | -3.3 (-9.1, 2.4) | 0.25 |
| Protein, % energy | 11.0 ± 1.0 | 11.2 ± 1.0 | 0.3 ± 1.4 | 0.39 | 11.6 ± 1.5 | 12.2 ± 0.1 | 0.6 ± 1.4 | 0.06 | 0.3 (-0.5, 1.1) | 0.45 |
| Saturated fat, g | 11.8 ± 5.0 | 12.4 ± 4.0 | 0.6 ± 4.5 | 0.54 | 11.0 ± 4.7 | 10.9 ± 4.6 | -0.1 ± 4.5 | 0.90 | -0.7 (-3.3, 1.9) | 0.59 |
| Saturated fat, % energy | 6.7 ± 2.6 | 6.6 ± 1.8 | -0.1 ± 2.4 | 0.85 | 6.5 ± 2.3 | 6.3 ± 1.8 | -0.1 ± 2.7 | 0.85 | -0.01 (-1.5, 1.5) | 0.99 |
| Monounsaturated fat, g | 9.8 ± 4.1 | 11.7 ± 7.9 | 2.0 ± 7.1 | 0.18 | 9.2 ± 4.5 | 9.0 ± 3.4 | -0.2 ± 2.7 | 0.67 | -2.2 (-5.3, 0.8) | 0.15 |
| Monounsaturated fat, % energy | 5.6 ± 1.9 | 6.0 ± 2.3 | 0.5 ± 1.9 | 0.25 | 5.2 ± 1.6 | 5.3 ± 1.0 | 0.1 ± 1.3 | 0.77 | -0.4 (-1.3, 0.6) | 0.42 |
| Polyunsaturated fat, g | 17.6 ± 5.9 | 19.3 ± 5.7 | 1.7 ± 4.8 | 0.09 | 17.3 ± 8.9 | 16.9 ± 5.8 | -0.4 ± 5.5 | 0.73 | -2.1 (-5.1, 0.9) | 0.16 |
| Polyunsaturated fat, % energy | 9.9 ± 2.3 | 10.5 ± 1.8 | 0.6 ± 2.0 | 0.19 | 9.6 ± 2.6 | 10.1 ± 1.6 | 0.4 ± 2.4 | 0.38 | -0.13 (-1.4, 1.2) | 0.84 |
| Sucralose, mg | - | 0.0 ± 0.0 | - | - | - | 13.1 ± 4.0 | - | - | 13.1 (11.5, 14.8) ^**^ | <0.0001 |

^*^Data presented as mean ± standard deviation. Dietary variables represent estimated daily intakes.

^**^Between-group difference in estimated sucralose intake at 12 weeks

**Supplemental Table 6.** Effect of the 12-week sucralose intervention on change in waist circumference (Δ waist circumference) or change in body weight (Δ body weight), adjusting for change in gut microbial features during the sucralose intervention to investigate potential sources of mediation

|  | **Δ waist circumference** | | | | **Δ body weight** | | | |
| --- | --- | --- | --- | --- | --- | --- | --- | --- |
|  | **β (SE)** | **p-value** |  |  | **β (SE)** | **p-value** |  |  |
| **Base model** | -0.64 (0.57) | 0.26 |  |  | -0.87 (0.43) | 0.05 |  |  |
| **+ Δ community composition** | **β (SE)** | **p-value** | **Proportion Mediated**  **(95% CI)** | **p-value** | **β** | **SE** | **Proportion Mediated (95% CI)** | **p-value** |
| Shannon Index | -0.52 (0.61) | 0.40 | 19 (-165, 284) | 0.54 | -0.82 (0.46) | 0.08 | 6 (-31, 114) | 0.67 |
| Simpson Index | -0.58 (0.60) | 0.34 | 9 (-225, 379) | 0.76 | -0.60 (0.44) | 0.18 | 32 (-16, 192) | 0.15 |
| Firmicutes:Bacteroides | -0.66 (0.60) | 0.28 | 0 (-64, 54) | 0.85 | -0.89 (0.45) | 0.05 | 2 (-14, 56) | 0.82 |
|  |  |  |  |  |  |  |  |  |
| **+ Δ species relative abundance** | **β (SE)** | **p-value** | **Proportion Mediated**  **(95% CI)** | **p-value** | **β (SE)** | **p-value** | **Proportion Mediated (95% CI)** | **p-value** |
| *Agathobacter* | -1.00 (0.66) | 0.14 | -56 (-629, 601) | 0.41 | -1.11 (0.50) | 0.03 | -28 (-265, 40) | 0.34 |
| *Fusicatenibacter* | -1.06 (0.63) | 0.10 | -65 (-575, 572) | 0.30 | -1.02 (0.49) | 0.04 | -17 (-143, 56) | 0.39 |
| *Megamonas* | -0.40 (0.63) | 0.53 | 38 (-341, 561) | 0.52 | -0.72 (0.48) | 0.14 | 17 (-79, 208) | 0.56 |
| *Enterococcus* | -0.76 (0.63) | 0.24 | -19 (-370, 336) | 0.65 | -0.75 (0.48) | 0.13 | 15 (-72, 173) | 0.66 |
| *Lachnoclostridium* | -0.77 (0.64) | 0.23 | -21 (-310, 233) | 0.66 | -0.94 (0.49) | 0.06 | -7 (-124, 84) | 0.73 |
| *Lachnospira* | -0.65 (0.62) | 0.30 | -1 (-245, 176) | 0.87 | -0.91 (0.47) | 0.06 | -5 (-68, 72) | 0.75 |
| *Lachnospiraceae NC2004 group* | -0.77 (0.61) | 0.21 | -20 (-213, 199) | 0.45 | -0.88 (0.46) | 0.07 | -0.5 (-73, 84) | 0.99 |
| *Anaerostipes* | -0.88 (0.60) | 0.15 | -37 (-376, 229) | 0.38 | -1.08 (0.45) | 0.02 | -24 (-173, 23) | 0.20 |
| *CAG-352* | -0.54 (0.61) | 0.38 | 15 (-197, 305) | 0.61 | -1.06 (0.46) | 0.02 | -22 (-194, 20) | 0.24 |
| *Roseburia* | -0.63 (0.61) | 0.31 | 1 (171, 179) | 0.99 | -0.89 (0.46) | 0.06 | -2 (-91, 88) | 0.89 |
| *Dorea* | -0.62 (0.62) | 0.33 | 4 (-229, 267) | 0.96 | -0.73 (0.47) | 0.13 | 16 (-51, 172) | 0.51 |
| *Lachnospiraceae UCG-004* | -0.54 (0.60) | 0.38 | 16 (-378, 221) | 0.72 | -0.70 (0.45) | 0.13 | 20 (-22, 114) | 0.27 |
| *Pediococcus* | -1.01 (0.61) | 0.10 | -58 (-439, 218) | 0.27 | -1.00 (0.47) | 0.04 | -15 (-140, 88) | 0.58 |
| *Faecalibacterium* | -0.82 (0.62) | 0.19 | -29 (-287, 286) | 0.47 | -0.98 (0.47) | 0.04 | -12 (-137, 94) | 0.62 |
| *Lachnospiraceae ND3007 group* | -1.04 (0.62) | 0.10 | -63 (-795, 469) | 0.30 | -0.96 (0.48) | 0.05 | -10 (-115, 62) | 0.65 |
| *Unclassified Lachnospiraceae* | -0.56 (0.62) | 0.37 | 13 (-260, 249) | 0.88 | -0.69 (0.46) | 0.14 | 21 (-63, 139) | 0.48 |

^*^P-values and β coefficients are derived from linear regression models examining the effect of the sucralose intervention on change in the specified outcome (Base model). Additional models adjust for change in gut microbiome community composition (Δ community composition) or individual species relative abundance (Δ species relative abundance).

**Supplemental Table 7.** Change in anthropometric and diet measures among study participants randomized into the sucralose intervention or control arm in a subset of participants with gut microbiome data in the overweight/obesity trial (n=48)^*^

| **Variables** | **Control group (n=25)** | | | | **Intervention group (n=23)** | | | | **Between-group difference in change**  **(95% CI)** | **Between-group**  **p-value** |
| --- | --- | --- | --- | --- | --- | --- | --- | --- | --- | --- |
|  | **Baseline** | **End of 12 weeks** | **Change** | **Within-group**  **p-value** | **Baseline** | **End of 12 weeks** | **Change** | **Within-group**  **p-value** |  |  |
| Body weight, kg | 71.2 ± 13.6 | 71.4 ± 13.8 | 0.1 ± 1.7 | 0.70 | 74.5 ± 11.1 | 74.9 ± 11.3 | 0.4 ± 1.9 | 0.33 | 0.3 (-0.8, 1.3) | 0.62 |
| Body mass index, kg/m^2^ | 28.4 ± 3.8 | 28.5 ± 3.8 | 0.03 ± 0.7 | 0.78 | 30.2 ± 4.8 | 30.4 ± 4.9 | 0.2 ± 0.8 | 0.35 | 0.1 (-0.4, 0.6) | 0.59 |
| Waist circumference, cm | 93.1 ± 8.8 | 92.9 ± 9.4 | -0.2 ± 2.1 | 0.67 | 95.4 ± 12.2 | 94.6 ± 12.4 | -0.8 ± 3.5 | 0.28 | -0.6 (-2.3, 1.0) | 0.46 |
| Total Energy, Kcal | 1649 ± 365 | 1663 ± 357 | 14 ± 304 | 0.83 | 1565 ± 355 | 1539 ± 273 | -25 ± 387 | 0.76 | -39 (-240, 163) | 0.70 |
| Carbohydrate, g | 235 ± 48.0 | 243 ± 48.8 | 7.9 ± 40.9 | 0.35 | 231 ± 55.5 | 219 ± 38.0 | -11.9 ± 60.2 | 0.35 | -19.7 (-49.4, 10.0) | 0.19 |
| Carbohydrate, % energy | 57.8 ± 6.2 | 59.6 ± 5.8 | 1.8 ± 4.4 | 0.05 | 60.1 ± 6.6 | 57.4 ± 3.6 | -2.7 ± 6.5 | 0.06 | -4.5 (-7.7, -1.3) | 0.007 |
| Total Sugar, g | 38.4 ± 15.9 | 39.8 ± 14.6 | 1.4 ± 15.2 | 0.64 | 27.7 ± 10.7 | 19.2 ± 5.9 | -8.5 ± 13.2 | 0.005 | -9.9 (-18.2, -1.6) | 0.02 |
| Total Sugar, % energy | 9.2 ± 2.9 | 9.4 ± 2.2 | 0.2 ± 3.5 | 0.78 | 7.1 ± 2.5 | 5.1 ± 1.6 | -2.1 ± 3.2 | 0.006 | -2.2 (-4.2, -0.3) | 0.02 |
| Added Sugar, g | 16.4 ± 12.5 | 16.1 ± 11.8 | -0.3 ± 16.6 | 0.92 | 18.1 ± 9.7 | 0.4 ± 2.1 | -17.6 ± 9.4 | <0.0001 | -17.3 (-25.2, -9.3) | <0.0001 |
| Added Sugar, % energy | 3.9 ± 3.1 | 3.5 ± 2.5 | -0.4 ± 3.8 | 0.60 | 4.4 ± 2.3 | 0.1 ± 0.5 | -4.3 ± 2.2 | <0.0001 | -3.9 (-5.8, -2.1) | <0.0001 |
| Fiber, g | 21.5 ± 5.2 | 24.2 ± 6.1 | 2.7 ± 5.2 | 0.02 | 23.2 ± 6.7 | 21.8 ± 3.5 | -1.4 ± 5.9 | 0.26 | -4.1 (-7.3, -0.9) | 0.01 |
| Protein, g | 52.1 ± 14.2 | 51.2 ± 13.1 | -0.9 ± 9.7 | 0.65 | 50.4 ± 13.0 | 47.5 ± 9.2 | -2.9 ± 12.2 | 0.26 | -2.0 (-0.84, 4.3) | 0.52 |
| Protein, % energy | 12.6 ± 1.6 | 12.3 ± 1.3 | -0.3 ± 1.5 | 0.32 | 12.8 ± 1.4 | 12.3 ± 1.0 | -0.5 ± 1.5 | 0.14 | -0.2 (-1.1, 0.7) | 0.68 |
| Saturated fat, g | 15.0 ± 7.4 | 15.9 ± 6.5 | 0.9 ± 6.6 | 0.51 | 12.3 ± 4.9 | 12.6 ± 4.9 | 0.3 ± 5.5 | 0.81 | -0.6 (-4.2, 2.9) | 0.73 |
| Saturated fat, % energy | 7.8 ± 2.9 | 8.5 ± 2.6 | 0.7 ± 3.1 | 0.26 | 6.9 ± 2.1 | 7.3 ± 2.7 | 0.3 ± 2.2 | 0.46 | -0.4 (-2.0, 1.2) | 0.63 |
| Monounsaturated fat, g | 12.6 ± 4.2 | 12.4 ± 4.4 | -0.2 ± 3.6 | 0.73 | 11.7 ± 4.2 | 10.5 ± 3.0 | -1.2 ± 4.7 | 0.24 | -0.9 (-3.3, 1.5) | 0.44 |
| Monounsaturated fat, % energy | 6.8 ± 1.3 | 6.5 ± 1.3 | -0.2 ± 1.3 | 0.41 | 6.6 ± 1.8 | 6.0 ± 0.9 | -0.6 ± 1.8 | 0.13 | -0.4 (-1.3, 0.5) | 0.40 |
| Polyunsaturated fat, g | 21.2 ± 5.9 | 21.9 ± 6.5 | 0.6 ± 5.5 | 0.58 | 20.6 ± 6.7 | 19.9 ± 6.0 | -0.7 ± 6.6 | 0.63 | -1.3 (-4.8, 2.2) | 0.47 |
| Polyunsaturated fat, % energy | 11.4 ± 1.6 | 11.7 ± 2.0 | 0.3 ± 2.1 | 0.50 | 11.7 ± 2.4 | 11.5 ± 1.8 | -0.2 ± 2.2 | 0.65 | -0.5 (-1.7, 0.7) | 0.42 |
| Sucralose, mg | - | 0.0 ± 0.0 | - | - | - | 13.0 ± 5.7 | - | - | 13.0 (10.8, 15.3) ^**^ | <0.0001 |

^*^Data presented as mean ± standard deviation. Dietary variables represent estimated daily intakes.

^**^Between-group difference in estimated sucralose intake at 12 weeks

**Supplemental Table 8.** Association of changes in added sugar intake with changes in gut microbial community and species among individuals in the type 2 diabetes sucralose intervention

| **Community composition** | **β** | **SE** | **p-value** |
| --- | --- | --- | --- |
| Shannon Index | 0.07 | 0.05 | 0.14 |
| Simpson Index | 0.004 | 0.03 | 0.45 |
| Firmicutes:Bacteroides | -0.03 | 0.11 | 0.77 |
|  |  |  |  |
| **Genus relative abundance** | **β** | **SE** | **p-value** |
| *Fusicatenibacter* | 1.71 | 0.51 | 0.001 |
| *Agathobacter* | 1.50 | 0.55 | 0.008 |
| CAG-352 | 0.71 | 0.30 | 0.02 |
| Lachnospira | 1.21 | 0.55 | 0.03 |
| *Dorea* | 0.84 | 0.39 | 0.03 |
| *Roseburia* | 1.10 | 0.51 | 0.04 |
| *Pediococcus* | -1.59 | 0.82 | 0.05 |
| *Faecalibacterium* | 1.06 | 0.57 | 0.07 |
| *Anaerostipes* | 0.88 | 0.50 | 0.08 |
| *Enterococcus* | -1.44 | 0.83 | 0.09 |
| *Lachnoclostridium* | 0.78 | 0.54 | 0.15 |
| Unclassified *Lachnospiraceae* | 0.39 | 0.30 | 0.20 |
| *Lachnospiraceae* NC2004 group | 0.38 | 0.50 | 0.44 |
| *Megamonas* | 0.17 | 0.76 | 0.82 |
| *Lachnospiraceae* ND3007 group | -0.05 | 0.52 | 0.99 |
| *Lachnospiraceae* UCG-004 | -0.03 | 0.36 | 0.99 |

^*^P-values and β coefficients for species are derived from the added sugar intake x time interaction in a linear mixed model including each gut microbial feature as the outcome, participant ID as a random-effect, and added sugar, time, and added sugar intake × time interaction as fixed effects.

**Supplemental Table 9.** Association of sucralose intake (mg/day) with changes in gut microbial community and species among individuals in the type 2 diabetes sucralose intervention

| **Community composition** | **β** | **SE** | **p-value** |
| --- | --- | --- | --- |
| Shannon Index | -0.05 | 0.02 | 0.04 |
| Simpson Index | -0.005 | 0.003 | 0.07 |
| Firmicutes:Bacteroides | 0.08 | 0.05 | 0.13 |
|  |  |  |  |
| **Genus relative abundance** | **β** | **SE** | **p-value** |
| *CAG-352* | -0.71 | 0.23 | 0.003 |
| *Agathobacter* | -1.29 | 0.43 | 0.004 |
| *Fusicatenibacter* | -1.12 | 0.39 | 0.005 |
| *Pediococcus* | 1.50 | 0.57 | 0.01 |
| *Enterococcus* | 1.48 | 0.58 | 0.01 |
| *Lachnospiraceae* NC2004 group | -0.75 | 0.36 | 0.04 |
| *Lachnoclostridium* | -0.76 | 0.39 | 0.05 |
| *Dorea* | -0.54 | 0.28 | 0.05 |
| *Megamonas* | -1.07 | 0.58 | 0.07 |
| Unclassified Lachnospiraceae | -0.38 | 0.22 | 0.08 |
| *Roseburia* | -0.65 | 0.40 | 0.11 |
| *Faecalibacterium* | -0.70 | 0.44 | 0.11 |
| *Anaerostipes* | -0.56 | 0.37 | 0.14 |
| *Lachnospira* | -0.39 | 0.41 | 0.35 |
| *Lachnospiraceae* UCG-004 | 0.06 | 0.25 | 0.82 |
| *Lachnospiraceae* ND3007 group | -0.01 | 0.37 | 0.98 |

^*^P-values and β coefficients are for species derived from the sucralose x time interaction in a linear mixed model including each gut microbial feature as the outcome, participant ID as a random-effect, and sucralose intake, time, and sucralose intake × time interaction as fixed effects.
